# *OPN* gene locus is associated with the risk of knee osteoarthritis: a case–control study

**DOI:** 10.1042/BSR20182023

**Published:** 2019-02-27

**Authors:** Houlai Shang, Yuedong Hao, Wenhao Hu, Xiaohui Hu, Qing Jin

**Affiliations:** 1 Department of Orthopaedics, The Affiliated Huaian No.1 People’s Hospital of Nanjing Medical University, Huaian 223300, Jiangsu, China; 2 Department of Operation and Anesthesiology, The Affiliated Huaian No.1 People’s Hospital of Nanjing Medical University, Huaian 223300, Jiangsu, China

**Keywords:** knee osteoarthritis, OPN, single nucleotide polymorphism

## Abstract

***Background/aims:*** Studies have demonstrated that osteopontin (OPN) was associated with the severity and development of knee osteoarthritis (OA). ***Methods:*** The purpose of this case–control study was to investigate the association between *OPN* gene rs11730582 polymorphism and knee OA risk in a Chinese population. Genotyping was analyzed using standard PCR and restriction fragment length polymorphism (PCR-RFLP). ***Results:*** The present study found that C allele or CC genotype of *OPN* gene rs11730582 polymorphism was related to decreased risk for knee OA. Furthermore, positive associations were obtained amongst the females, and body mass index (BMI) < 25 kg/m^2^ groups. ***Conclusions:*** To sum up, the present study reveals that *OPN* gene rs11730582 polymorphism decreases the risk of knee OA in Chinese Han population.

## Introduction

Osteoarthritis (OA), one of the most prevalent degenerative joint diseases, is a major cause of disability in the elderly worldwide. Approximately 10% of the world’s population (9.6% of males and 18% of females) aged over 60 years have symptomatic OA, characterized by pain, limitation of joint activity, and deformity [1]. Knee OA, with the highest prevalence amongst all the sites, brought intolerable pain to up to 6% of all adults and often made a surgical intervention necessary [2]. Biochemical, biomechanical, molecular, and morphological changes in both cells and extracellular matrix (ECM) lead to a progressive cartilage loss, osteophyte formation, synovitis as well as degeneration of the joint [3]. Certain factors such as trauma, joint injury, increased age, weight and physical activity, may contribute to the development of OA [4]. OA is a complex disorder caused by the interaction of multiple genetic and environmental factors [5]. Several previous studies found that polymorphisms in certain genes had effects on the pathogenesis of OA [6–8].

Osteopontin (OPN) is a non-collagenous bone matrix protein produced by various cells, including chondrocytes, fibroblasts, epithelial and endothelial cells, osteoblasts, synoviocytes as well as tumor cells [9]. OPN serves as a biochemical marker of inflammation and a participant in some physiologic and pathologic processes, such as wound healing, bone turnover, tumorigenesis, and immune responses [10,11]. OPN played a pivotal role in secretion levels of interleukin (IL) 10 (IL-10), IL-3, IL-12, IFN-γ, NF-kB, regulating the osteoclast function and affecting CD44 receptors [10]. Studies have reported that some physiological processes like bone remodeling and cartilage-to-bone transitions was related to OPN expression [12].

*OPN* gene, located at 4q21-25, comprises seven exons and phosphorylated acidic glycoprotein codes in several tissues and cells. The OPN protein function could be affected by some polymorphic variants located in the transcriptional factor binding-site region [13,14]. The rs11730582 is located in the promoter region of *OPN* gene, which may affect transcriptional activity of OPN. It causes base changes (T to C). The association between *OPN* gene polymorphisms and OA risk was investigated previously [15,16]. However, the results were inconsistent. Thus, we designed this case–control study in a Chinese population to address the relationship between OPN gene polymorphism and the risk of knee OA.

## Materials and methods

A total of 403 knee OA patients 526 controls were selected from the Huaian First People’s Hospital. Primary OA was diagnosed as any symptom and sign of OA as well as radiographic signs of OA based on the Kellgren–Lawrence grade. We excluded the individuals with knee diseases, such as rheumatoid arthritis, post-traumatic arthritis, skeletal dysplasia, or developmental dysplasia. Controls were recruited from the individuals receiving the regular health checkup in the same hospital at the time of sample collection. The clinical characteristics including sex, age, body mass index (BMI), and Kellgren–Lawrence grading were extracted from medical records. The research has been carried out in accordance with the World Medical Association Declaration of Helsinki, and all subjects provided written informed consent. The study protocol was approved by the Institutional Review Board of Huaian First People’s Hospital.

Blood samples (3 ml) were drawn from the participants and genomic DNA was extracted using the TIANamp Blood DNA kit (Tiangen Biotech, Beijing, China) according to manufacturer’s instructions. The concentration and purity of the extracted DNA was measured by taking absorbance and running electrophoresis, respectively. Standard PCR and restriction fragment length polymorphism (PCR-RFLP) was used to genotype the selective SNP (*OPN* gene rs11730582 polymorphism) and the primers are presented as follows: 5′-CATGGATGAGGGAACAAGGATA-3′ (forward) and 5′-CATGGATGAGGGAACAAGGATA-3′ (reverse). PCR products were separated on 2% agarose gel and DNA was visualized by Ethidium Bromide (Invitrogen, Grand Island, U.S.A.) staining. Two independent investigators conducted the genotype analysis in a blind manner. Approximately 10% of randomly selected samples were selected randomly for confirmation, and the results were 100% consistent.

Comparisons of continuous variables and categorical variables were carried out using the Student’s *t* test and Chi-squared (χ^2^) test, respectively. Odds ratios (ORs) and 95% confidence intervals (CIs) calculating by logistic regression analysis were used to evaluate whether rs11730582 polymorphism was associated with the risk of knee OA. Five genetic models were used in the present study: the allele model (C vs. T), the dominant model (TC+CC vs. TT), the recessive model (CC vs. TT+TC), the codominant model (CC vs. TT), and the heterozygous model (TC vs. TT). The Hardy–Weinberg equilibrium (HWE) amongst controls was tested by a goodness-of-fit χ^2^ test. All statistical analyses were performed using the SAS software package (ver. 9.1.3; SAS Institute, Cary, NC, U.S.A.).

## Results

A total of 403 cases and 526 controls were enrolled in the present study. There were no significant differences in sex, age, BMI, alcohol, and smoking status for case and control groups. Specific clinical and demographic data are listed in Table 1. Mean age was 57.34 ± 8.85 years in cases and 57.88 ± 9.29 years in controls. The percentage of female groups were 65.8 and 68.3%, respectively.

**Table 1:** Patient demographics and risk factors in OA

Variable	Cases (*n*=403)	Controls (*n*=526)	*P*
Age (years)	57.34 ± 8.85	57.88 ± 9.29	0.372
Sex			0.422
Male	138 (34.2%)	167 (31.7%)	
Female	265 (65.8%)	359 (68.3%)	
Smoking			0.662
Yes	118 (29.3%)	161 (40.0%)	
No	285 (70.7%)	365 (60.0%)	
Alcohol			0.920
Yes	153 (38.0%)	198 (37.6%)	
No	250 (62.0%)	328 (62.4%)	
BMI	26.83 ± 3.39	26.77 ± 3.47	0.768
Kellgren–Lawrence grading			
1	30 (7.4%)	-	-
2	175 (43.4%)	-	-
3	118 (29.3%)	-	-
4	80 (19.9%)	-	-

Table 2 delineated the genotype distributions and allele frequencies of *OPN* gene polymorphisms in OA patients and control subjects. Genotype distributions for rs11730582 polymorphism in the controls conformed to the HWE (*P*=0.957). Logistic regression analyses revealed that CC genotype was associated with a decreased risk of OA compared with TT genotype (CC vs. TT, OR: 0.67; 95% CI: 0.45–0.99; *P*=0.048). Additionally, *OPN* gene rs11730582 polymorphism decreased the risk of knee OA under the allelic model (Table 2). Moreover, significant associations were observed amongst the females, and BMI < 25 kg/m^2^ groups (Table 3).

**Table 2:** Logistic regression analysis of associations between polymorphisms and risk of OA

Genotype	Cases* (*n*=403)		Controls* (*n*=526)		OR (95% CI)	*P*
	*n*	%	*n*	%		
rs11730582 T/C						
TT	165	40.9	183	34.8	1.00	
TC	184	45.7	254	48.3	0.80 (0.61–1.07)	0.130
CC	53	13.2	89	16.9	**0.67 (0.45–0.99)**	**0.048**
TC+CC	237	58.8	343	65.2	0.77 (0.59–1.01)	0.054
TT+TC	349	86.6	437	83.1	1.00	
CC	53	13.2	89	16.9	0.75 (0.52–1.09)	0.134
T allele	514	63.9	620	58.9	1.00	
C allele	290	36.1	432	41.1	**0.81 (0.67–0.98)**	**0.029**

Bold values are statistically significant (*P*<0.05).

* The genotyping was successful in 402 cases and 526 controls for rs11730582.

**Table 3:** Stratified analyses between rs11730582 polymorphisms and the risk of OA

Variable	rs11730582 (Case/control)			TC vs. TT	CC vs. TT	CC vs. TT+TC	CC+TC vs. TT
	TT	TC	CC				
Sex							
Male	56/64	59/71	23/32	0.95 (0.58–1.56); 0.839	0.82 (0.43–1.57); 0.550	0.84 (0.47–1.52); 0.573	0.91 (0.57–1.44); 0.686
Female	109/119	125/183	30/57	0.75 (0.53–1.05); 0.096	**0.59 (0.35–0.98); 0.041**	0.69 (0.43–1.11); 0.128	**0.71 (0.51–0.98); 0.040**
Smoking							
Yes	44/51	55/81	18/29	0.79 (0.46–1.34); 0.375	0.75 (0.36–1.53); 0.421	0.71 (0.45–1.12); 0.138	0.78 (0.47–1.28); 0.321
No	121/132	129/173	35/60	0.81 (0.58–1.14); 0.228	0.64 (0.39–1.03); 0.067	0.86 (0.45–1.64); 0.641	0.77 (0.56–1.06); 0.103
Alcohol							
Yes	65/68	72/100	16/30	0.75 (0.48–1.18); 0.223	0.56 (0.28–1.12); 0.100	0.65(0.34–1.25); 0.199	0.71 (0.46–1.09); 0.120
No	100/115	112/154	37/59	0.84 (0.58–1.20); 0.333	0.73 (0.45–1.20); 0.217	0.81 (0.52–1.27); 0.357	0.81 (0.58–1.14); 0.220
Age (years)							
<55	63/61	68/96	20/32	0.69 (0.43–1.10); 0.116	0.63 (0.32–1.21); 0.164	0.77 (0.42–1.42); 0.407	0.67 (0.43–1.05); 0.079
≥55	102/122	116/158	33/57	0.88 (0.62–1.25); 0.474	0.69 (0.42–1.15); 0.152	0.74 (0.47–1.18); 0.210	0.83 (0.59–1.16); 0.274
BMI							
<25	52/52	56/77	7/32	0.73 (0.43–1.22); 0.226	**0.23 (0.09–0.56); 0.001**	**0.27 (0.11–0.70); 0.003**	**0.58 (0.36–0.96); 0.033**
≥25	113/131	128/177	46/57	0.84 (0.60–1.18); 0.308	0.94 (0.60–1.49); 0.778	1.03 (0.68–1.58); 0.886	0.86 (0.63–1.19); 0.362

Bold values are statistically significant (*P*<0.05).

## Discussion

In the present study, we explored the association between *OPN* gene polymorphism and knee OA risk in a Chinese case–control study and found that the *OPN* gene rs11730582 polymorphism may serve as a protective factor in the development of knee OA.

OPN, as one of the cytokines and cell attachment proteins, was shown to facilitate recovery from organism injury or infection [17]. The role of *OPN* gene rs11730582 polymorphism has been investigated in various diseases, such as intestinal metaplasia [18], nephrolithiasis [19], and diabetic nephropathy [20]. No significant association was observed between *OPN* rs11730582 polymorphism and risk of intestinal metaplasia [18] or nephrolithiasis [19]. However, Cheema et al. [20] found that this polymorphism was associated with a decreased risk for diabetic nephropathy in type 2 diabetic patients. We hypothesized that *OPN* rs11730582 polymorphism has disease-dependent functionality. Despite the role of *OPN* polymorphism in various diseases, little is known about their association with OA susceptibility.

OPN was an intrinsic inhibitor of IL-1, NO, and other inflammatory substances [21], thereby resulting in the suppression of the inflammatory process in cartilage. Honsawek et al. [22] indicated that OPN in plasma and synovial fluid was associated with progressive joint damage in knee OA. In addition, they showed OPN may serve as a biochemical marker for determining OA disease severity [22]. Two studies [15,16] explored the association between *OPN* gene rs11730582 polymorphism and knee OA risk, but their findings were conflicting. Lack of association was observed in a Mexican population between this SNP and knee OA risk [16]. However, Jiang et al. [15] found the *OPN* gene variants were related to the OA risk and the radiographic severity. In this study, we found that C allele or CC genotype of rs11730582 polymorphism in *OPN* gene decreased the risk of knee OA. Subgroup analyses observed positive findings in the female, and BMI < 25 kg/m^2^ groups, suggesting that these individuals were more likely to expose to these protective factors. Considering the limited sample sizes of subgroup analyses, interpreting these results with caution is needed. So how *OPN* gene rs11730582 polymorphism contributed to the decreased risk for OA? Matsui et al. [23] found that both ageing-associated and instability-induced OA was exacerbated in OPN knockout mice model, indicating a protective role of OPN in the development of OA. Furthermore, Attur et al. [21] reported that OPN inhibited the inflammation in cartilage via reduction in IL-1, NO, and PGE_2_ production. These studies provided compelling evidence that OPN was a protective factor for OA. We hypothesized that rs11730582 polymorphism may decrease the risk of knee OA by altering the OPN protein level, which warrants further studies to validate it.

Potential limitations of the present study should be considered. First, the relationship between the *OPN* gene rs11730582 polymorphism and knee OA susceptibility could not be fully revealed by a single case–control study as the sample size was not large enough, which might underpower the facticity. Second, the cases and controls were selected from hospitals, which may not serve as the exact representative of the general population. Third, functional analyses were absent to further discuss how the *OPN* gene polymorphisms affect knee OA. What is more, the distinction of right or left knee was not taken into consideration, but it has been reported that knee OA was prone to develop on the right knee amongst male patients [24]. Last but not the least, we evaluated the effects of *OPN* gene polymorphisms only amongst this Chinese population, which may induce selection bias to the whole ethnic groups.

In summary, *OPN* gene rs11730582 polymorphism is associated with decreased risk for knee OA. Subgroup analyses show that this SNP decreases the risk of knee OA in the female, and BMI < 25 kg/m^2^ groups However, this finding should be further confirmed by better-designed studies with larger sample sizes and ethnically diverse populations.

## Abbreviations

BMI: body mass index
CI: confidence interval
CD44: CD44 molecule
HWE: Hardy–Weinberg equilibrium
IFN-γ: interferon-γ
IL: interleukin
NF-kB: nuclear factor kappa B
NO: nitric oxide
OA: osteoarthritis
OR: odds ratio
PGE_2_: prostaglandin E_2_
SNP: single nucleotide polymorphism
